# Diagnostic sensitivity of 2-day cardiopulmonary exercise testing in Myalgic Encephalomyelitis/Chronic Fatigue Syndrome

**DOI:** 10.1186/s12967-019-1836-0

**Published:** 2019-03-14

**Authors:** Maximillian J. Nelson, Jonathan D. Buckley, Rebecca L. Thomson, Daniel Clark, Richard Kwiatek, Kade Davison

**Affiliations:** 1 Alliance for Research in Exercise, Nutrition and Activity (ARENA), School of Health Sciences, University of South Australia, GPO Box 2471, Adelaide, SA 5001 Australia; 2 Adelaide Medical School and Robinson Research Institute, University of Adelaide, Adelaide, SA Australia; 3 Division of Medicine, Lyell McEwin Hospital, Adelaide, SA Australia

**Keywords:** Chronic Fatigue Syndrome, Myalgic Encephalomyelitis, Ventilation, Biomarkers, Fatigue, Exercise

## Abstract

**Background:**

There are no known objective biomarkers to assist with the diagnosis of Myalgic Encephalomyelitis/Chronic Fatigue Syndrome (ME/CFS). A small number of studies have shown that ME/CFS patients exhibit an earlier onset of ventilatory threshold (VT) on the second of two cardiopulmonary exercise tests (CPET) performed on consecutive days. However, cut-off values which could be used to differentiate between ME/CFS patients have not been established.

**Methods:**

16 ME/CFS patients and 10 healthy controls underwent CPET on a cycle-ergometer on 2-consecutive days. Heart rate (HR), ventilation, ratings of perceived exertion (RPE) and work rate (WR) were assessed on both days.

**Results:**

WR at VT decreased from day 1 to day 2 and by a greater magnitude in ME/CFS patients (*p *< 0.01 *group* × *time* interaction). No interaction effects were found for any other parameters. ROC curve analysis of the percentage change in WR at VT revealed decreases of − 6.3% to − 9.8% provided optimal sensitivity and specificity respectively for distinguishing between patients with ME/CFS and controls.

**Conclusion:**

The decrease in WR at VT of 6.3–9.8% on the 2nd day of consecutive-day CPET may represent an objective biomarker that can be used to assist with the diagnosis of ME/CFS.

## Background

ME/CFS is a chronic condition of unexplained onset, characterised by both physical and mental fatigue, muscle and joint pain, and increased levels of post-exertional malaise when compared with healthy individuals [1]. The prevalence of ME/CFS has been estimated at 0.8 to 3.3% of the population [2]. The condition represents a significant challenge to patients and healthcare providers given that many patients are unable to maintain their occupation and there is no widely accepted treatment [3].

In addition to the lack of an accepted treatment for ME/CFS, difficulties also exist with its diagnosis. Many studies have attempted to identify a single, objective bio-marker to aid with diagnosis (e.g. [4–6]), however, to date, none has been identified. As a result, diagnosis has been performed on the basis of clinical criteria which aim to confirm a set of core symptoms and exclude other factors which might otherwise explain these symptoms. These clinical criteria have typically required the presence of fatigue exacerbated by exercise, sore throat, headaches, and unrefreshing sleep, among other symptoms [1, 3]. The 1994 Centre for Disease Control and Prevention (CDC) criteria [1] was the first widely accepted of these clinical criteria, and required patients to present with clinically evaluated, unexplained, persistent or relapsing fatigue persistent for 6 months. The fatigue reported by the patient must be: of new or definite onset; not the result of ongoing exertion; not substantially alleviated by rest; and results in substantial reduction in previous levels of occupational, educational social or personal activities. Additionally, the fatigue must be accompanied by four or more of the following symptoms: impaired short term memory or concentration; sore throat; tender cervical or axillary lymph nodes; muscle pain; multi-joint pain without arthritis; headaches of a new type, pattern, or severity; unrefreshing sleep; and post-exertional malaise lasting more than 24 h [1]. This clinical criteria was followed by the 2003 ‘Canadian Consensus Criteria’ [3] which required patients to meet the criteria for fatigue, post exertional malaise and/or fatigue, sleep dysfunction and pain, together with two or more neurological/cognitive manifestations and one or more symptoms from two of the categories of autonomic, neuroendocrine and immune manifestations, with the illness required to have persisted for at least 6 months [3]. The most recent widely used clinical criteria is the 2011 ‘International Consensus Criteria’ [7], which requires a number of pathological neurological impairments, immune/gastro-intestinal/genitourinary impairments, and energy metabolism/ion transport impairments, with an increased focus on the requirement for patients to demonstrate post-exertional malaise as a key feature of the condition [7] in an attempt to better differentiate ME/CFS from other similar conditions (e.g. fibromyalgia).

In line with the requirement by the 2011 International Consensus Criteria for patients to demonstrate post-exertional malaise, physiological differences between ME/CFS patients and healthy controls following physical exertion have been investigated [8, 9] as potential biomarkers of the condition. In particular, the effects of cardiopulmonary exercise tests (CPET) performed over 2 consecutive days (2-day CPET) have been evaluated in an attempt to identify post-exertional, fatigue-induced biomarkers that can discriminate between ME/CFS patients and controls [10–13]. Multiple studies [8, 10, 11, 14] have identified that ME/CFS patients experience an earlier onset of VT on the 2nd day of the 2-day CPET protocol, a change that is not present in healthy controls. Although this earlier onset of VT may represent an objective biomarker of ME/CFS, results have been inconsistent in relation to the magnitude of change in the onset of VT (ranging from negligible [12], to large [8]). However, although requiring patients with ME/CFS to complete graded exercise tests to exhaustion on 2 consecutive days may exacerbate symptoms, and is therefore not ideal for assisting with the diagnosis of ME/CFS, due to the lack of reliable and objective biomarkers to aid in the diagnosis of ME/CFS, 2-day CPET represents an increasingly popular method for attempting to differentiate between patients and controls [13]. Indeed, ME/CFS management practices [13] and legal processes (e.g. insurance claims [15]) are being based on this reported phenomenon, despite the current research being limited to a small number of studies, indicating further confirmation of these findings is required.

Presently, despite the increasing use of 2-day CPET to differentiate between patients and controls, further evidence is needed to confirm the usefulness of this technique. Additionally, in previous studies no diagnostic cut-off values have been established for the change in the onset of VT which limits its validity in clinical practice. Establishing such values would allow for the confirmation of the ability for 2-day CPET to reliably differentiate between ME/CFS patients and healthy controls, and therefore potentially be useful for assisting with the diagnosis of ME/CFS. Accordingly, this study had two aims: (1) to determine if 2-day CPET is able to differentiate between ME/CFS patients and healthy controls, and (2) to establish cut-off values which can be used in differentiating between ME/CFS patients and healthy controls.

## Methods

### Participants

Sixteen ME/CFS patients were recruited via specialist clinics and ME/CFS support groups from the Adelaide, South Australia Greater Metropolitan area. Ten healthy participants were recruited to act as controls using convenience sampling from patient and research centre networks. Controls were matched to ME/CFS patients on the basis of age, body mass index (BMI) and physical activity status. All participants were required to be between the ages of 18–65 years, and ME/CFS patients had to have been previously diagnosed with ME/CFS based on one of three widely accepted diagnostic criteria: (1) 1994 Centers For Disease Control and Prevention (CDC 1994—also known as the ‘Fukuda’ criteria [1]), (2) 2003 ‘Canadian’ Consensus Criteria (CCC) [3], or (3) 2011 International Consensus Criteria (ICC) [7]. With the exception of the patients being diagnosed with ME/CFS, all participants had to self-report as free of additional health conditions and injuries, and were required to be classed as low-moderate risk based on a self-report pre-exercise health screening [16] — all self-report screenings were conducted in the presence of an Accredited Exercise Physiologist, and any potential participants who were classified as moderate risk based on this screening process underwent additional screening to ensure that participation in the study represented minimal risk to the participant. All participants were required to be sedentary (< 150 min of moderate physical activity per week) and were excluded if they were taking any medication or had any known medical conditions (excluding ME/CFS) which could alter the response to exercise (e.g. beta-blockers, anti-depressants/postural orthostatic tachycardia syndrome). Experimental procedures were approved by the University of South Australia Human Research Ethics Committee and all participants provided written informed consent prior to participating.

### Experimental procedures

#### Familiarisation

Participants first attended the laboratory for an initial habituation session, during which they were familiarised with the laboratory and with the questionnaires and procedures to be used during the study. Participants first completed the Chalder Fatigue Scale, an 11 item questionnaire which measures the severity of fatigue, and has been previously validated in ME/CFS patients [17], before undergoing familiarisation with the exercise protocols to be used within the study. Given that ME/CFS patients typically experience a significant exacerbation of their symptoms following strenuous physical exertion, it was not feasible to expose participants to the full protocol used with the main testing sessions (which included a maximal exercise test) within the familiarisation session. Instead, participants were familiarised with the exercise equipment by performing 5 min of submaximal exercise on an electronically-braked cycle ergometer (Ergoselect 200, Ergoline GmbH, Bitz, Germany) while heart rate (HR) was measured using a HR monitor (RS800CX, Polar Electro Oy, Kempele, Finland), and were connected to an indirect calorimetry system (TrueOne 2400, Parvo Medics, East Sandy, Utah) via a two-way non-rebreathing value (Hans-Rudolph inc., Shawnee, Kansas). Throughout all testing sessions, efforts were made to minimise the amount of time that participants spent in waiting areas, and were given access to a reclining chair while waiting according to the recommendations of Stevens et al. [13].

#### 2-day CPET protocol

Following the familiarisation session, participants returned to the laboratory at least 24 h later for the first of two exercise testing sessions, which consisted of a submaximal warm-up followed by CPET. Each session had an identical protocol and were performed on consecutive days. Participants first completed the Chalder Fatigue Scale before being fitted with the HR monitor and then resting supine for 10 min. Participants were then seated on the bicycle ergometer, fitted with the breathing valve for the indirect calorimetry system, and told to rest quietly while sitting on the bike. Following a seated rest period of 4–6 min, participants were instructed to commence cycling at a self-selected cadence for 5 min at 40 W for males and 30 W for females, which served as a warm-up. Following the initial 5 min of steady state exercise, the work rate was increased by 5 W increments every 20 s, until volitional exhaustion. All participants were given frequent verbal encouragement throughout the incremental portion of the test, to help elicit a maximal effort [18]. Immediately following the cessation of exercise, participants were assisted to dismount the cycle ergometer and lay supine for 2 min. Ratings of perceived exertion (RPE) were collected at the end of each minute during the exercise test using Borg’s 6–20 RPE scale [19]. Following the initial exercise test, participants returned to the laboratory at the same time on the following day, and the protocol was repeated in an identical fashion. Following each maximal exercise test, all participants were monitored whilst resting within the laboratory until they felt well enough to leave of their own accord. Additionally, participant well-being was monitored over the 2-weeks following the testing, to ensure no adverse events.

### Outcome measures

Peak oxygen uptake (peak V̇O_2_) was defined as the highest value for any 15-s epoch obtained during the exercise test. Determination of peak V̇O_2_ is typically done by identifying a plateau in V̇O_2_ in response to successive increments in workload, however this response is difficult to obtain in sedentary subjects [20]. As a result, V̇O_2_ values were considered to have reached a valid maximal level if participants fulfilled two or more secondary criteria: (1) achievement of at least 90% of age predicted maximal HR [21], (2) respiratory exchange ratio (RER) > 1.1, and (3) RPE ≥ 17 [22]. Participants’ data were excluded from the analysis if they were unable to produce a maximal effort based on these criteria. VT was calculated using the V-Slope method [23]. Ventilatory data used for calculation of VT consisted of the final 30 s of steady state workload data, and all subsequent ventilatory data points.

All HR data were downloaded as R–R intervals to Polar Protrainer 5 software (Polar Electro Oy, Kempele, Finland), where artefacts or ectopic heartbeats were removed using the software’s automatic data filtering function. Resting HR (RHR) was defined as the average HR during the final 2 min of the pre-exercise supine rest. Steady state HR (SSHR) was defined as the average HR during the final 30 s of the 5-min steady state exercise which preceded the incremental portion of the testing. Peak HR was defined as the maximum HR produced during the maximal exercise test.

### Statistical analysis

Statistical analysis was performed using IBM SPSS Statistics 21 (IBM Corporation, Armonk, NY). All data were checked for normality of distribution using the Shapiro–Wilk test prior to analysis. Unpaired t-tests were used to determine if there were any differences at baseline (Day 1) between patients and controls for any dependant variables. To determine the effect of post-exertional malaise on the dependant variables, two-way repeated measures ANOVA was performed to identify any main effects of *group* (patient or control), *time* (day 1 or day 2) or any *group* × *time* interaction effects. Where significant main effects were identified, estimated marginal means were assessed to determine where those differences occurred. To determine if any of the assessed parameters represent a useful tool to aid in differentiating between ME/CFS patients and controls, receiver operator characteristic (ROC) analysis was conducted on any objective physiological variables which demonstrated a significant *group* or *group* × *time* interaction in order to compute sensitivity and specificity of these variables for differentiating ME/CFS participants from controls. Statistical significance was set at *p *< 0.05. All data are presented as mean ± standard deviation (SD).

## Results

Participant physical characteristics are shown in Table 1. There were no differences in any physical characteristics between the ME/CFS patients and controls. ME/CFS patients had been diagnosed with the condition for 8.1 ± 4.7 years, with durations ranging from 2 to 18 years.

**Table 1:** Participant characteristics

	Controls	ME/CFS	p-value
n (female/male)	10 (5/5)	16 (9/7)	–
Age (years)	49.8 (13.7)	50.3 (12.5)	0.46
Height (m)	1.69 (0.07)	1.71 (0.11)	0.87
Body mass (kg)	70.5 (9.8)	76.3 (18.5)	0.37
BMI (kg/m^2^)	24.6 (3.0)	25.9 (5.3)	0.50

Data are mean (SD)

*ME/CFS* Myalgic Encephalomyelitis/Chronic Fatigue Syndrome, *n* sample size, *m* metres, *kg* kilograms, *BMI* body mass index

Ventilatory, subjective and HR parameters from the 2-day CPET are provided in Table 2. All participants met at least two of the three criteria required for determination of a valid maximal effort during exercise testing. ME/CFS patients reported higher scores than controls on the Chalder Fatigue Scale on Day 1 (*p *< 0.01), with a significant *group* × *time* interaction effect following Day 2, due to a greater increase in their scores compared with controls (*p *< 0.01). Significant effects of *time* for HR at VT (*p *= 0.03), WR at VT (*p *< 0.01) and RER at VT (*p *= 0.02) were also identified, with estimated marginal means indicating that all three parameters were lower on Day 2 irrespective of group. Significant *group* effects were found for RPE (*p *< 0.01), and RPE at VT (*p *< 0.01), with ME/CFS patients having higher values for both. Importantly, there was a significant *group* × *time* interaction for WR at VT (*p *< 0.01), with WR at VT decreasing by a greater extent from Day 1 to Day 2 for ME/CFS patients compared with healthy controls.

**Table 2:** Subjective, heart rate and ventilatory parameters obtained during consecutive days of maximal exercise testing

Variable	Controls Day 1	ME/CFS Day 1	Controls Day 2	ME/CFS Day 2	‘Group’ effect p-value	‘Time’ effect p-value	‘Group’ × ‘Time’ interaction p-value
Chalder Fatigue Score	11.2 (0.6)	20.3 (6.8)	11.2 (0.6)	25.3 (7.0)	*<* *0.001*	*0.005*	*0.005*
Resting							
HR (bpm)	69.5 (8.4)	74.0 (12.1)	70.1 (10.9)	72.0 (12.0)	0.47	0.60	0.35
VO_2_ (ml/kg/min)	4.1 (0.8)	4.5 (1.4)	4.1 (0.7)	4.1 (1.3)	0.57	0.46	0.49
Steady state (30 W for female, 40 W for male)							
HR (bpm)	100.6 (11.1)	106.5 (12.6)	101.0 (12.7)	104.5 (14.2)	0.31	0.71	0.57
VO_2_ (ml/kg/min)	11.6 (1.3)	11.4 (1.3)	11.4 (1.3)	11.3 (2.1)	0.80	0.49	0.83
RPE	8.3 (1.6)	10.5 (1.9)	7.5 (1.7)	10.4 (2.3)	*0.001*	0.32	0.40
Ventilatory threshold							
HR (bpm)	122.4 (13.2)	124.0 (18.3)	120.8 (14.2)	117.4 (17.6)	0.89	*0.03*	0.17
WR (watts)	90.5 (17.1)	87.8 (29.6)	88.0 (16.7)	72.5 (27.7)	0.37	*<* *0.001*	*0.003*
VO_2_ (ml/kg/min)	16.5 (2.0)	15.9 (4.1)	15.9 (1.5)	15.4 (3.4)	0.69	0.08	0.82
RPE	10.8 (1.9)	13.0 (1.4)	10.7 (1.6)	12.6 (2.0)	*0.003*	0.41	0.63
Maximal exercise							
HR (bpm)	170.3 (10.0)	167.8 (20.0)	170.9 (11.0)	167.0 (15.7)	0.60	0.93	0.66
WR (watts)	172.0 (35.5)	154.4 (56.0)	174.0 (36.6)	152.5 (51.7)	0.32	0.97	0.27
VO_2_ (ml/kg/min)	29.9 (6.1)	27.3 (9.2)	30.3 (6.2)	27.4 (8.8)	0.40	0.54	0.73
RPE	16.4 (1.4)	17.5 (1.4)	17.0 (1.6)	17.6 (1.3)	0.11	0.20	0.40

Values are mean (SD), Italic text indicates p-value < 0.05

*ME/CFS* Myalgic Encephalomyelitis/Chronic Fatigue Syndrome, *HR* heart rate, *VO*_2_ volume of oxygen uptake, *ml* millilitres, *kg* kilogram, *min* minute, *RPE* rating of perceived exertion, *WR* work rate

**Fig. 1:**
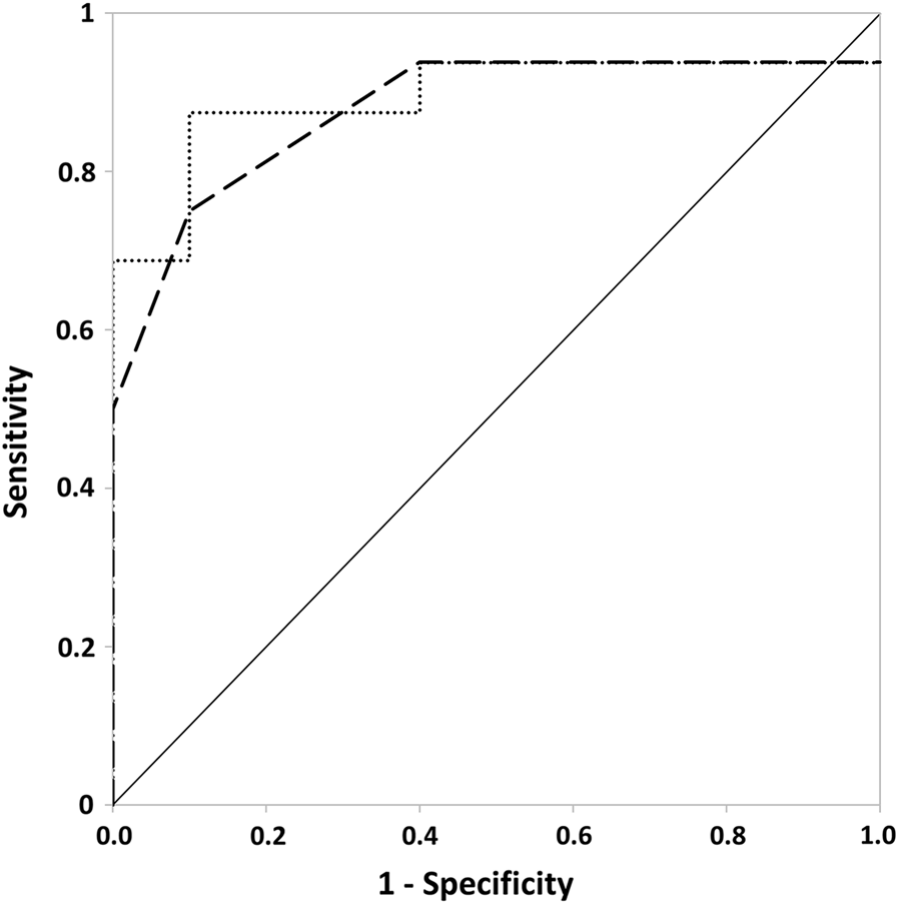
Receiver operator characteristic (ROC) curves for absolute and percentage change in work rate at ventilatory threshold from Day 1 to Day 2 of testing. *Dashed line* absolute change, *dotted line* percentage change, *solid line* indicator of matched specificity and sensitivity

Receiver operator characteristic analysis of absolute and percentage changes in WR at VT (Fig. 1) showed an area under the curve of 87.8% for the absolute change (*p *< 0.01, standard error: 7.1%), and 89.4% for the percentage change (*p *< 0.01, standard error: 6.8%). Absolute change data revealed that greater absolute and percentage reductions in WR at VT occurred in patients with ME/CFS compared with controls, with thresholds for optimal sensitivity and specificity for distinguishing between ME/CFS and controls represented by an absolute difference in reduction in WR at VT of between 7.5 W (sensitivity = 75%, specificity = 90%) and 12.5 W (sensitivity = 50%, specificity = 100%). For percentage changes in WR at VT, sensitivity and specificity were optimal at differences in reductions of between − 6.3% (sensitivity = 87.5%, specificity 90%) and − 9.8% (sensitivity = 68.8%, specificity = 100%). For the percentage change in WR at VT, 12 out of the 16 patients experienced a decrease in this parameter of greater than 10%, while such a change was only observed in one control participant.

## Discussion

This study aimed to (1) determine if 2-day CPET is a useful method for differentiating between ME/CFS patients and healthy controls, and (2) establish cut-off values which can be used to aid in the diagnosis of ME/CFS. The main finding of the present study was that during the 2nd day of the 2-day CPET, WR at VT was decreased for ME/CFS patients, confirming previous findings that an earlier onset of VT during the presence of post-exertional malaise is a potential biomarker of ME/CFS [8, 10, 11]. The change in WR at VT was paired with larger self-reported increases in fatigue in ME/CFS patients compared with controls on Day 2, while there were no differences in peak WR or peak VO_2_ from Day 1 to Day 2 for either patients or controls. ROC analysis identified that the decrease in WR at VT of 6.3–9.8% on the 2nd day of 2-day CPET may represent an objective biomarker of ME/CFS, with high sensitivity and specificity.

In the current study, there were no differences between patients and controls for RHR, SSHR, peak HR, peak V̇O_2_ or peak WR, suggesting that the patients and controls were well matched for their general fitness levels. Previous literature has suggested that ME/CFS patients may be affected by significant deconditioning compared to healthy, sedentary individuals [24], or kinesiophobia [25, 26], which may impact on their ability to produce a maximal effort. However, given the similarities between the patients and controls for peak V̇O_2_, SSHR, peak HR, and that all ME/CFS patients were able to produce a criteria-defined valid maximal effort, this does not appear to be the case in the current study. These findings are in agreement with the those of Sargent et al. [27] who reported no difference in V̇O_2_ max between ME/CFS patients and controls when accepted criteria were applied to define the attainment of a maximal value. Importantly, the fact that ME/CFS patients produced the same WR and V̇O2 as controls on both days of testing implies that the change in any ventilatory or HR parameters as a result of the 2-day CPET were not due to a change in physical performance in the ME/CFS patients.

Receiver operator characteristic analysis performed on the change in WR at VT between Day 1 and Day 2 of testing revealed that an absolute decrease in WR at VT of 7.5–12.5 W or a percentage decrease of between − 6.3% and − 9.8% provided optimal specificity and sensitivity for differentiating between patients and controls. However, given the large variation in WR at VT for ME/CFS patients (ranging from 50 to 140 W in the current study), it is likely more appropriate to use the percentage, rather than absolute change in VT, as a method for differentiating between the two groups. It is important to acknowledge that CPET may exacerbate symptoms of post-exertional malaise and is therefore not an ideal method for determining the presence of ME/CFS, and markers that can be assessed under resting conditions would be preferable. However, while not ideal, evaluation of the change in WR at VT during 2-day CPET may be a valid and sensitive marker that can be used to aid in the diagnosis of ME/CFS. Based on the ROC analysis performed in the present study, we would propose that the more conservative measure of at least a 9.8% reduction in WR at VT be used for diagnosis, given this provided 100% specificity, indicating that a reduction of this magnitude is not likely to occur in a person who does not have ME/CFS.

Numerous previous studies have also found an earlier onset of VT in ME/CFS patients on the 2nd day of 2-day CPET. VanNess, Snell and Stevens [10] found a 30% reduction in V̇O2 at VT from the first to the 2nd day, while Keller, Pryor and Giloteaux [11] found a 15% decrease in V̇O_2_ at VT and a 21% decrease in WR at VT, Snell et al. [8] found a 55% decrease in WR at VT and Hodges, Nielsen and Backen [14] found a 12% decrease in WR at VT for ME/CFS patients while controls experienced a 9% increase. While these results suggest that ME/CFS patients experience an earlier occurrence of VT on the 2nd day of 2-day CPET, the mechanism responsible for this effect is currently unknown. Potentially, the alterations in VT during the 2-day CPET may result from metabolic abnormalities. Metabolic abnormalities have been reported in skeletal muscle of ME/CFS patients related to impairment of oxygen delivery to skeletal muscle during exercise and inability to recover from exercise-induced reductions in pH [28–30]. Derangement of pH homeostasis might have resulted in a more rapid decrease in blood pH during the 2nd day of exercise in the present study. This would in turn lead to an increased CO_2_ production through buffering of H^+^ ions by carbonic anhydrase, leading to stimulation of chemoreceptors with a subsequent increase in ventilation. However, while some studies have reported derangement of pH homeostasis in patients with ME/CFS, others have found such abnormalities are present in less than 50% of patients [31, 32]. In the present study though, VT occurred earlier in all but one ME/CFS patients (i.e. in 15/16 patients). Keller et al. [11] found a decrease in maximal O_2_ pulse during the 2nd day of their 2-day CPET, indicating a compromised oxygen delivery in ME/CFS patients in the presence of post-exertional malaise, while Vermeulen et al. [12] found a non-significant 5% decrease in the same parameter on the 2nd day of testing. A lack of O_2_ delivery/uptake may lead to an earlier transition to anaerobic energy systems and therefore a more rapid increase in lactic acid production and reduction in pH. Future studies employing a 2-day maximal testing protocol in patients with ME/CFS should endeavour to measure lactic acid and pH. Interestingly, the change in WR at VT from Day 1 to Day 2 in the current study was smaller than that seen in previous studies which have assessed this parameter in ME/CFS patients [8, 10, 11]. This is likely due to a difference in the protocols used to elicit a maximal effort. In the current study all participants were given regular verbal encouragement to help elicit a valid maximal effort. Conversely, it was not explicitly stated if verbal encouragement was provided in two of the three pervious studies [10, 11], and it has been shown that frequent verbal encouragement results in higher peak WR’s and prolonged maximal exercise tolerance [18] than when no encouragement is provided. This potential lack of encouragement may have resulted in a premature cessation of exercise on Day 2 of the 2-day CPET in ME/CFS patients, resulting in a greater exacerbating in the change in WR at VT.

This study is limited by a possible selection bias as a result of the 2-day CPET protocol used within the study. Given the potential for symptom exacerbation as a result of the 2-day CPET, patients with severe ME/CFS may be less likely to volunteer, whereas patients with mild-moderate ME/CFS may be more likely. Anecdotally, none of the included participants classified themselves as a severe sufferer of the condition, and so the inclusion of patients with a more severe form of ME/CFS may have produced a different result. Future research should attempt to include sufferers with severe ME/CFS in the study design; however the authors acknowledge the potential for severe symptom exacerbation for these people and hence the clinical and ethical considerations. Additionally, ME/CFS patients exhibited a wide range of time periods since initial diagnosis (ranging from 2 to 18 years). This may have affected the results as patients who have been suffering from the condition for longer periods may experience increased levels of physical detraining that are common in ME/CFS patients compared to those who had been diagnosed more recently.

## Conclusion

ME/CFS patients exhibit a decrease in WR at VT on the 2nd day of 2-day CPET, which was accompanied with increases in self-reported increases in fatigue that were not present in healthy controls. Importantly, the decreases in WR at VT was not accompanied by any change in performance on the maximal exercise test on Day 2 of testing (as demonstrated by unchanged peak HR and peak VO2), indicating that the change in WR at VT in ME/CFS may represent an objective biomarker of the condition, with a decrease in WR at VT of at least 9.8% providing the greatest specificity for distinguishing between ME/CFS patients and healthy controls. Future research should attempt to determine the mechanism for the earlier onset of VT in ME/CFS patients.

## Abbreviations

ANOVA: analysis of variance
BMI: body mass index
CCC: Canadian Consensus Criteria
CDC: Centres for Disease Control and Prevention
CFS: Chronic Fatigue Syndrome
CPET: cardiopulmonary exercise test
HR: heart rate
ICC: International Consensus Criteria
ME: Myalgic Encephalomyelitis
RER: respiratory exchange ratio
RHR: resting heart rate
RPE: rating of perceived exertion
ROC: receiver operator characteristic
SSHR: steady state heart rate
V̇O_2_: volume of oxygen consumption
VT: ventilatory threshold
W: watts
WR: work rate
